# Hydrogen-Assisted Aging Applied to Storage and Sealing Materials: A Comprehensive Review

**DOI:** 10.3390/ma16206689

**Published:** 2023-10-14

**Authors:** A. K. M. Ahsanul Habib, Ahmed Nazmus Sakib, Zarin Tasnim Mona, Md Monjur Hossain Bhuiyan, Pejman Kazempoor, Zahed Siddique

**Affiliations:** 1 Department of Materials Science and Engineering, Rajshahi University of Engineering & Technology, Rajshahi 6204, Bangladesh; ahsanulhabibsoron@gmail.com; 2 Department of Aerospace and Mechanical Engineering, University of Oklahoma, Norman, OK 73019, USA; zarin@ou.edu (Z.T.M.); mhbhuiyan@ou.edu (M.M.H.B.); pkazempoor@ou.edu (P.K.); zsiddique@ou.edu (Z.S.)

**Keywords:** hydrogen, aging, polymer, storage and sealing materials, sustainability, pipeline, leak

## Abstract

Hydrogen is a possible alternative to fossil fuels in achieving a sustainable energy future. Unlike other, older energy sources, the suitability of materials for storing, distributing, and sealing systems in a hydrogen environment has not been comprehensively studied. Aging, the extended exposure of a material to an environmental condition, with hydrogen causes degradation and damage to materials that differ from other technologies. Improved understanding of the physical and chemical mechanisms of degradation due to a gaseous hydrogen atmosphere allows us to better select and develop materials that are best suited to carrier and sealing applications. Damage to materials from aging is inevitable with exposure to high-pressure hydrogen. This review discusses the specific mechanisms of different categories of aging of storage and sealing materials in a hydrogen environment. Additionally, this article discusses different laboratory test methods to simulate each type of aging. It covers the limitations of current research in determining material integrity through existing techniques for aging experiments and explores the latest developments in the field. Important improvements are also suggested in terms of material development and testing procedures.

## 1. Introduction

### 1.1. Prospects and Limitations of Hydrogen Energy in Terms of Aging

Conventional energy sources, such as fossil fuels, are extensively used to meet global energy demands. However, the combustion of these fuels has been shown to contribute to greenhouse effects, global warming, acid rain, and other environmental issues. These issues have long been a concern for environmental sustainability. Hydrogen, being the most abundant element in the universe and a clean energy source, is a promising energy storage vector for supporting a sustainable energy future. Using hydrogen to produce energy is advantageous to fossil fuels because the primary byproducts of the process are energy, heat, and water. As a result, different environmental issues still need to be minimized if hydrogen is to replace current energy sources [1]. Another advantage of hydrogen is its high specific energy density; it can provide approximately three times as much energy per unit of mass as conventional fuels such as diesel or gasoline, i.e., approximately 120 MJ/kg [2]. Therefore, hydrogen fuel cell vehicles, as opposed to traditional transportation systems, may be a sustainable choice because they can bring about a reduction in greenhouse gas emissions, eliminate fuel feedstock discrepancy, and improve onboard fuel efficiency [3]. A comparison of the energy storage capacity of different fuels with hydrogen is summarized in Table 1.

With these benefits of hydrogen in mind, we must begin to look at the complications of using hydrogen as an energy source. The ability of hydrogen to dissolve in and through metals can result in leakage and material failure. We consider these the most significant dangers of dealing with hydrogen. A gas cloud is created when hydrogen leaks into the air, and if it encounters an ignition source, hydrogen cloud explosions are likely to occur [5]. Material failure due to hydrogen can take place in different forms, such as hydrogen-induced cracking (HIC), hydride embrittlement (HE), hydrogen-induced blistering, and other detrimental effects, that commonly lead to catastrophic fractures [6]. Long-term exposure to a hydrogen environment increases the degradation and damage rate of many materials in a process known as aging. Under normal conditions, the degradation and damage rates of many materials are far slower compared to what is observed under long-term exposure to a hydrogen environment. Thus, it is a great challenge for the hydrogen industry to understand the aging processes and to try to find remedies. In one study, researchers created a catalyst by enclosing palladium nanoparticles in a special dendrimer called PAMAM. They tested different dendrimer variations and found that the best catalyst, with G6 generation, amine end groups, and a Jeffamine core (P6.NH2), efficiently produced hydrogen from ammonia borane at room temperature. This finding could advance hydrogen storage for clean energy applications, as it enables safe and efficient hydrogen release at lower temperatures using well-designed catalysts [7]. The service life and safety performance of materials used in the hydrogen industry are therefore dependent on taking the aging process into consideration during the design phase [8]. 

### 1.2. History of Hydrogen-Induced Aging

Almost half of the pipeline infrastructure still in service in the United States is 40 or more years old. Older pipelines, particularly those that have been in service for several decades, are often made of steel. “Aging” refers to the continual time-dependent degradation of materials under normal operations [8]. Numerous factors, including temperature, creep, fatigue, and brittleness, lead to the aging phenomenon in a hydrogen environment. According to a database of Dutch-investigated accidents, 25% of accidents are related to aging through material deterioration [9]. The aging phenomenon can be categorized into material deterioration, obsolescence, and organizational aging. The discussion in the current article is limited to material deterioration [10]. Some historical incidents that have taken place due to aging are discussed in Table 2.

### 1.3. Impact of Operating Conditions on Hydrogen-Assisted Aging

Operating conditions have a significant effect on hydrogen-induced aging. This effect manifests itself as expedited deterioration of metallic components after several years of exposure. The result shows that the hydrogen expedited the deterioration process. Slobodyan et al. [17] took into consideration, for instance, the top and bottom parts of the pipe that were used in an oil pipeline for 30 years. Residual water that was left over after the transported hydrocarbons acted as an aggressive species, accumulated at the pipe’s bottom, and created an environment that was conducive to corrosion and subsequent hydrogenation of the bottom section of the pipe. Thus, the lower half of the pipe had significantly more defective steel characteristics than the top. Continuous exposure of pipeline systems to challenging operating circumstances might hasten the aging process and result in unheard-of breakdowns from hydrogen-induced damage. When certain materials are exposed to hydrogen and stress at the same time, this can cause a serious problem called hydrogen embrittlement. This can lead to increased risk of failure from internal and external stresses due to the increased brittleness of the material. Atomic hydrogen may be produced through the adsorption and dissociation of hydrogen gas on steel pipeline surfaces [18,19]. A material failure mode is introduced, and several damage processes are linked to the entry of atomic hydrogen into interstitial spaces. According to ASME B31.12, pipes must adhere to specified requirements to be used in service lines for gaseous and liquid hydrogen [20].

### 1.4. Potential Reasons of Hydrogen-Assisted Aging

The tiny hydrogen molecules are impressive flee artists, which is the main distinction between hydrogen-driven aging and other liquids in the same specimen. In general, the low boiling point (−253 °C), lower density in the gaseous stage (0.09 kg/NA m^3^), and high density in the liquid phase (70.9 kg/NA m^3^) of hydrogen present challenges for conventional storage facilities [21]. Not only can hydrogen diffuse through solid steel, but it can also travel through the slightest of seal flaws. Elastomeric materials are typically utilized extensively in sealing components as well as other applications, such as control valves, liners, connectors, and flexible hoses, which are also subjected to high-pressure hydrogen under operating conditions [3]. Rubbery O-rings are a sealing component that can blister, a type of internal fracture where damage takes place due to the rapid decompression of highly pressurized hydrogen gas [22]. The entry of atomic hydrogen into interstitial gaps results in leakage. Thus, the minimal size and the density difference in different physical states make it a very challenging material for storage. Additionally, its high diffusivity through metallic and polymeric materials makes it a challenging material for sealing. It has a high tendency to leak as well. 

### 1.5. Potential Mechanism of Hydrogen-Induced Aging on Storage and Sealing Materials

**Figure 1:**
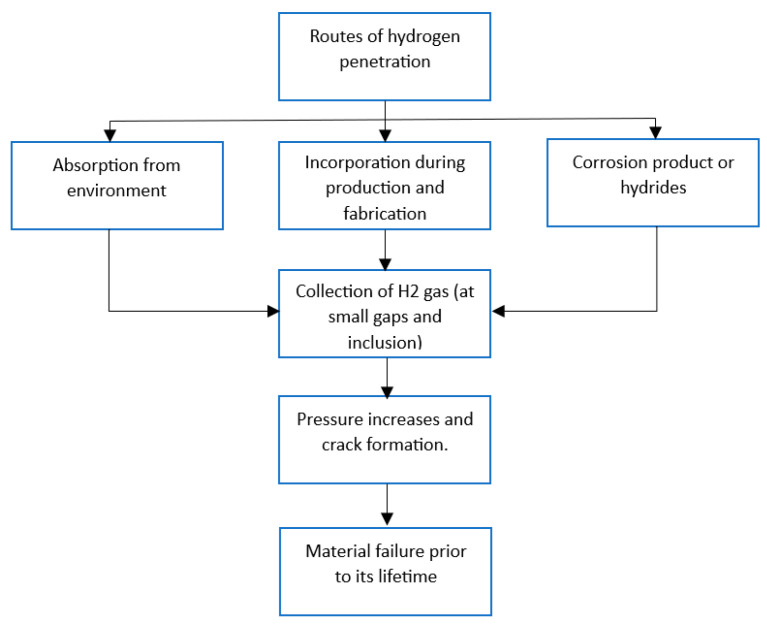
Hydrogen-induced aging mechanism flowchart [20,23].

Hydrogen-induced aging of storage and sealing materials can occur through several mechanisms. One potential mechanism is hydrogen diffusion into the material, where the hydrogen atoms can react with and weaken the chemical bonds within the material. This can cause a loss of elasticity and increased stiffness, leading to cracks and failures over time. A simple flowchart of the hydrogen-induced aging mechanism is shown in Figure 1.

The associated reactions are below [15,18]:

H^+^ + e^−^ → H_ads_. (hydrogen atom)

H_ads_ + H_ads_ → H_2_ (hydrogen molecule)

The cumulative effects of process operations demonstrates that residual deformation can frequently occur without observable structural component damage. Laboratory-scale research is needed to obtain a detailed analysis of likely event data and evaluate whether hydrogen-induced aging is a substantial concern for a particular application, in addition to identifying the key elements or problems that are causing it. In this review article, the aging phenomenon of hydrogen storage and sealing materials will be discussed, along with the various types of aging and their mechanisms in a hydrogen environment. Alongside this, a comparison of the various laboratory tests that have been carried out to understand the aging process and predict the service life of the materials will be discussed as well. The goal of the review is to identify research shortcomings to be further investigated with the intention of contributing to standard aging research and protocols

## 2. Methods of Aging in a Hydrogen Environment

**Figure 2:**
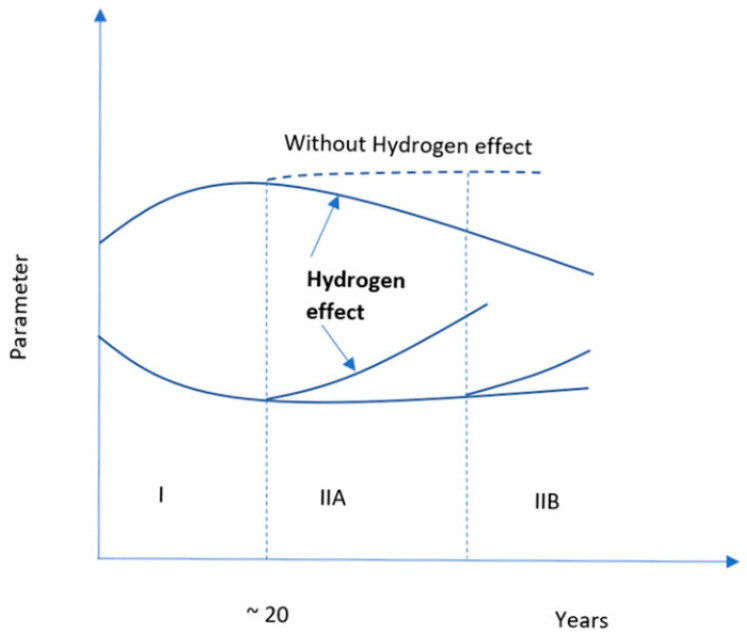
Two-stage degradation of materials in a hydrogen environment [24].

Because materials degrade more quickly in hydrogen environments, materials used for storage and sealing applications must account for this degradation. The general patterns of operating deterioration of the properties of infrastructure materials, as depicted in Figure 2, were summarized by Nykyforchyn et al. [24].

Figure 2 indicates the degradation of materials over time in a hydrogen environment. Stage I represents aging by deformation, and Stage II contains two stages, IIA and IIB, which represent dispersed damage and the buildup of damage in the rolling direction, respectively. In Stage II, due to the accumulation of hydrogen, microdamage develops, which erodes the natural integrity of the material. In Stage II, the dotted line represents the behavior of the materials in the absence of hydrogen. Thus, it indicates that hydrogen can accelerate the degradation process of the materials [24,25]. Hydrogen embrittlement is an example of degradation that follows the trend shown in Figure 2. The incorporation of hydrogen in metals creates a secondary hydride phase, which is brittle. As a result of brittleness, the structural integrity of the metals becomes weak, which degrades the metal more rapidly than in normal conditions.

Therefore, the theme supports the aging process demonstrated in the figure. However, there is still significant disagreement over the main mechanism by which this degradation process occurs. Adsorption-induced dislocation emission (AIDE), hydrogen-enhanced localized plasticity (HELP), and the hydrogen-enhanced de-cohesion mechanism (HEDE) are the fundamental mechanisms that that are believed to be responsible for hydrogen-induced embrittlement [26]. Determining the correct materials for the storage and sealing of hydrogen is critical in combatting these degradation mechanisms and for hydrogen to be used as an energy source because productivity drastically declines if produced hydrogen cannot be stored appropriately. Materials can be aged in a hydrogen environment through various processes, including thermal aging, chemical aging, mechanical aging, mechanical–chemical aging, and thermo-mechanical aging.

### 2.1. Thermal Aging

Thermal aging of materials is the permanent structural, compositional, and morphological changes over time while exposed to service temperatures. Materials can structurally degrade because of thermal cycling, which can accelerate the diffusion processes of hydrogen. The solubility of hydrogen in metals is proportional to temperature. With decreasing temperatures, diffusion mobility decreases [27]. Therefore, thermal aging increases with increasing temperatures.

#### 2.1.1. Mechanism of Thermal Aging

**Figure 3:**
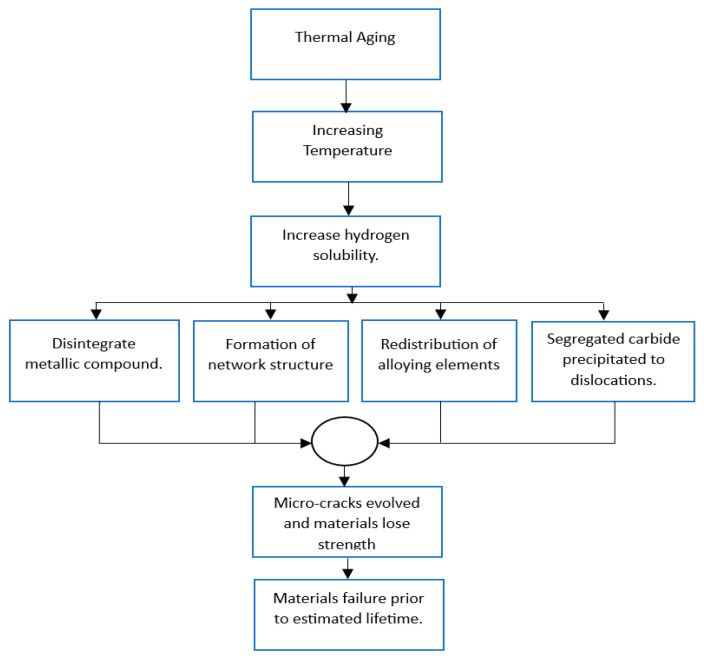
Flowchart of the mechanism of thermal aging [29,30].

Hydrogen thermal aging is directly related to the diffusion of hydrogen at different temperatures. Sharp temperature drops can decrease the equilibrium hydrogen solubility in metals [28]. At 570 °C and 100 °C, Kolachev [25] found that the coefficient of hydrogen diffusion was 2 × 10^−4^ cm^2^/s and 4.4 × 10^−5^ cm^2^/s, respectively, which indicates the co-efficient of hydrogen diffusion varied with temperature. The flowchart in Figure 3 represents the mechanism of thermal aging.

#### 2.1.2. A Laboratory Test for Thermal Aging

For the thermal aging of structural steel under a hydrogen environment, Student O.Z. [23] used a hydrogen-filled chamber to expose a specimen at 0.3 MPa of pressure to cyclic temperature changes between 100 °C and 570 °C at a rate of 100 °C /min with a pause between temperature direction changes. After cooling at 100 °C at a rate of 50 °C/min, the 12Kh1MF steel specimen was kept in a vacuum during the thermal cycle for two hours. It has been observed that due to the temperature variation, the equilibrium solubility of hydrogen changes, which leads to the disintegration of cementite and the network formation of alloyed carbides because of thermal cycling, thermal stresses evolved and created microcracks, which caused the thermal aging of structural steel [31]. Hirakami et al. [32] conducted a laboratory test with two samples of drawn pearlitic steel that were aged at 100 °C and 300 °C for 10 min and tested under a slow strain rate testing method for measuring the hydrogen embrittlement susceptibility. The result shows that the hydrogen embrittlement susceptibility of the steels at 300 °C is reduced because the carbons are segregated from the dislocations and the carbides are precipitated from the dislocations. Age-hardened beryllium–copper alloy, a suitable replacement for austenitic stainless steel used to store hydrogen at refueling stations, was tested in a laboratory by Ogawa et al. [33]. They observed that the hydrogen solubility capacity of the beryllium–copper alloy was two to three times lower than that of austenitic stainless steel. However, after exposing the specimen to 100 MPa hydrogen for 500 h at high temperatures of 270 °C, hydrogen diffusion may be enhanced. Consequently, the specimen’s mechanical qualities declined; for instance, only the tensile strength fell by approximately 5% [33,34].

The thermal aging of sealing materials has not been extensively studied yet. Yamabe et al. [34], Fujiwara et al. [35], and Simmons et al. [36] tested elastomeric sealing material NBR at room temperature, 30 °C, and 110 °C, and found that there is no hydrogenation or structural activity below 30 °C, but at 110 °C, compression set is increased by 40%, which indicates that with increasing temperature, the elastomeric sealing materials show a thermal aging tendency. Menon et al. [37], Castagnet et al. [38], and Klopffer et al. [39] tested different types of thermoplastic polymeric sealing materials at room temperature and 20–80 °C, respectively, and observed some random behavior. The degree of crystallinity can be increased with increasing temperature, but mechanical properties do not change. This is because, with increasing temperatures, the plasticization of the polymer also increase. This phenomenon indicates that these thermoplastics can resist aging to some extent.

### 2.2. Chemical Aging

Chemical aging occurs when materials react with the chemicals in the environment they are exposed to over a period of time. This reaction causes permanent changes in structure, composition, and morphology. In this method, materials remain in contact with different chemicals, mostly immersed in the solution and charged with hydrogen [36,37,40,41]. This type of aging is frequently seen by both storage and sealing materials due to their continuous interaction with chemicals.

#### 2.2.1. Mechanism of Chemical Aging

**Figure 4:**
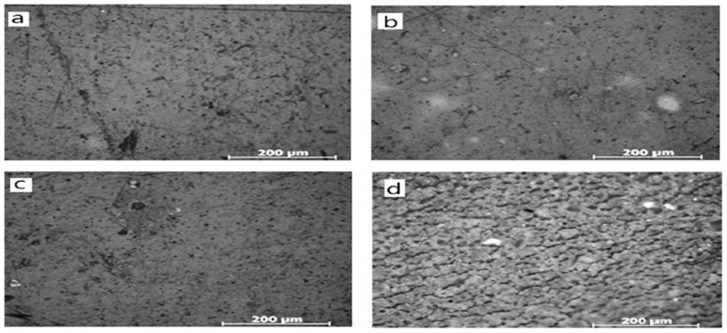
Micromorphology of samples exposed to test solutions: (**a**) unexposed, (**b**) exposed to mild conditions for sufficient time, (**c**) unexposed, and (**d**) exposed to harsh conditions for sufficient time [40].

Chemical aging is one of the most common forms of aging found in hydrogen storage and sealing materials. In hydrogen-storing metallic materials, the hydrogen’s distribution and state influence the hydrogen embrittlement and the tensile properties of the metal and lead to complete aging [37,42]. In hydrogen-sealing materials, cracks are formed by the decomposition of the backbone and the hydrolysis of crosslinks, which leads to aging [40]. This phenomenon can be observed in Figure 4.

**Figure 5:**
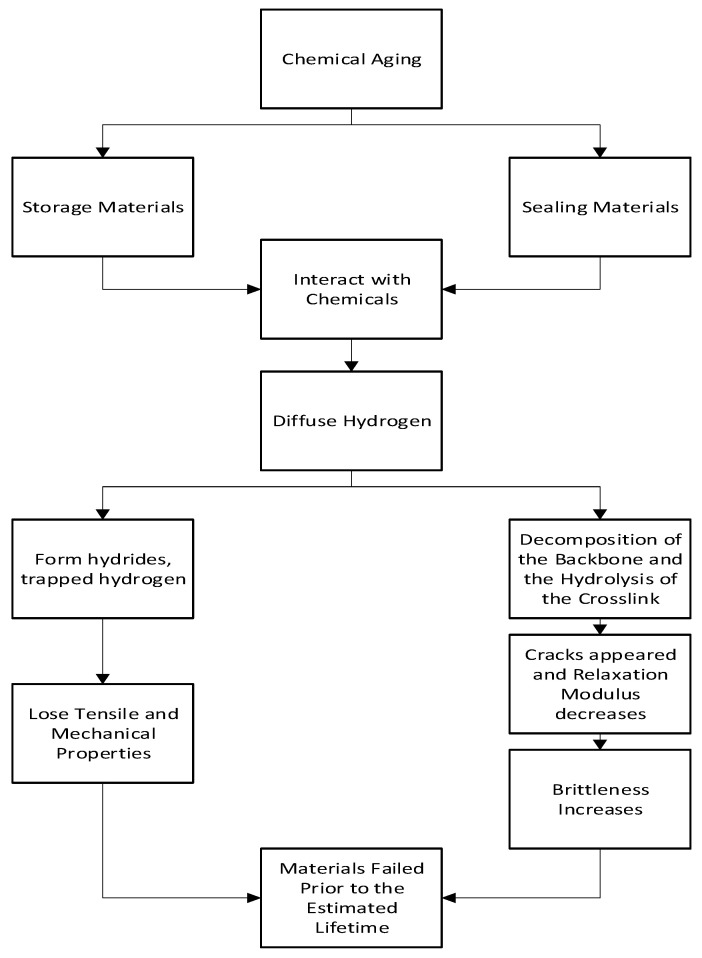
Flowchart of the mechanism of chemical aging [37,40].

The micromorphological analysis conducted by Li et al. [40] mimics the surface of the sealing material. It shows that with increasing hydrogen content, the condition becomes harsher, and cracks appear. Figure 4a,c are unexposed samples; Figure 4b,d are exposed to mild and harsh conditions, respectively. From this figure, it is clear that as the harshness of the condition increases, the surface roughness also increases. Surface roughness indicates the formation of cracks here. Thus, surface roughness indicates the aging of polymeric sealing materials. Figure 5’s flowchart indicates the chemical aging mechanism for hydrogen storage and sealing materials.

#### 2.2.2. The Laboratory Test for Chemical Aging

Ogawa et al. [37] used martensitic Ni-Ti alloy specimens and acidulated phosphate fluoride (APF) solutions for the aging test. Cathodic electrolysis was used for charging the samples with hydrogen, and the charged specimens were aged for 16 h in the air at room temperature. The results show that the reduction in tensile strength increased brittleness and led the sample toward aging. Ogawa et al. [37] further conducted a different type of post-aging test on non-immersed and immersed specimens to determine the post-aging behavior of the material, where a tensile test was performed within a few minutes after the removal of the specimen from the solution at room temperature. The results show that the tensile strength of the sample can be decreased. A Vickers microhardness test was performed to determine the hardness of the specimen. Results show that the hardness can be increased, which increases the brittleness. TDA and XRD analyses were performed to check the corrosion products and hydrides on the surface and for the quantitative analysis of trapping hydrogen, respectively. Each test confirmed the pick for hydrogen absorption and hydride formation, indicating the aging was being processed after charging.

Tal-Gutelmacher et al. [38] conducted laboratory tests on three Titanium-based alloys: Ti-6Al-4V, Beta-21S, and Ti-20wt%Nb. The Ti-6Al-4V alloy was thermo-mechanically treated and exposed to a hydrogen environment. The alloy formed brittle titanium hydroxide phases, which is a key factor in chemical aging. Beta-21S was exposed to hydrogen electrochemically at room temperature and shows good resistance to hydrogen.

Ti-20 wt.% Nb was exposed to hydrogen in two ways: electrochemically and in a gaseous environment. Electrochemical exposure formed (Ti, Nb) Hx hydride, and gaseous exposure created only titanium hydride. In both cases, Ti-20 wt.% Nb had to face chemical aging. Nykyforchyn et al. [35] tested ferrite-perlite X52 steel pipe after long-term service at the gas trunkline and found that all the mechanical properties of the material deteriorated due to the increment of hydrogen trapping. The bottom side of the pipe severely deteriorated, indicating that during the flow of aggressive substances through the pipe, the maximum portion of hydrogen penetrated the pipe and was trapped there. OMURA et al. [39] conducted a laboratory test on stainless steel and Ni-based alloys and found that high-nitrogen stainless steel and Ni alloy 286 had the maximum hydrogen concentration (Hc) at which fracture elongation was hard to degrade. High-nitrogen stainless steel had a higher Hc than the concentration of hydrogen-absorbing HE in the service environment, lowering the chance of hydrogen embrittlement in the service condition. For internally reversible hydrogen embrittlement, alloys 286 and 718 show severe results compared to the cathodic charge.

Li et al. [43] conducted a laboratory test using elastomeric gaskets made of silicone rubber as specimens. Different concentrations of acid were used for the test. Depending on the concertation, one solution was called a regular solution, which has an environment similar to a regular working environment, and another one was highly concentrated and used for an accelerated durability test (ADT). The concentration for the regular solution was 12.5 ppm sulfuric acid and 1.8 ppm hydrofluoric acid, and the concentration for ADT was 1 mil. L^−1^ sulfuric acid and 30 ppm hydrofluoric acid. During the experiment, the decomposition of the backbone and the hydrolysis of the crosslink of the polymeric gasket material take place, leading to a reduction in the elasticity and sealing force. As a result, the material aged in a hydrogen environment. KLOPFFER et al. [44] tested polyethylene and polyamide membranes and tubes under a hydrogen environment with controlled pressure and temperature. After long-term exposure to hydrogen, the measured permeability capacity of polyamide became lower than that of polyethylene. However, the mechanical properties of both materials remain almost unchanged.

### 2.3. Mechanical Aging

The mechanical aging of the materials is a slow, progressive, and irreversible process of losing the capability to perform the assigned work under a high mechanical loading condition. Mechanical aging is often observed in fuel cell conditions where high-pressure hydrogen molecules create a high loading condition on the sealing material and in the hydrogen storage system. 

#### 2.3.1. Mechanism of Mechanical Aging

**Figure 6:**
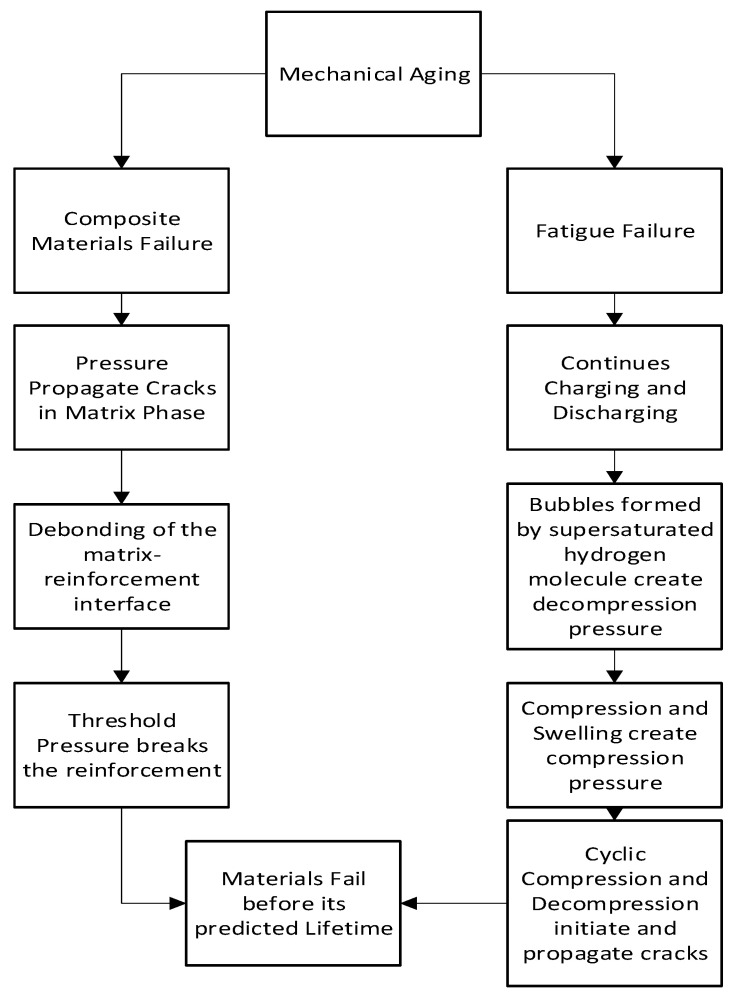
Flowchart of the mechanism of mechanical aging [35,43].

Mechanical aging is found in hydrogen storage tanks made of composite materials. Burst pressure, fiber damage, fatigue, collapse, and blistering of liners are some failure modes in this type of aging. Figure 6 flowchart indicates the mechanism of mechanical aging. 

When pressure is applied to a composite material, cracks can develop and extend profoundly to the matrix phase, which facilitates interface debonding. When the pressure reaches its threshold, a substantial quantity of reinforcement fails and causes the material to implode. On the other hand, continuous charging and discharging cause mechanical fatigue, which impacts mechanical aging. The mechanism for the formation of these types of cracks involves the initiation and growth of cracks as a result of strain concentration. The strain concentration can be caused by supersaturated hydrogen molecules creating bubbles after decompression and strain because of compression and swelling. Shear stress, induced by the concentration gradient of solute gas molecules, initiates randomly organized circumferential cracks. As compressive tension increases so does volume expansion or swelling. As a result, tensile stress increases and fracture propagation accelerates. When the decompression rate is accelerated, cracks in the surface appear visibly [35,43].

#### 2.3.2. The Laboratory Test for Mechanical Aging

Wang et al. [45] analyzed the carbon fiber/epoxy composite used in hydrogen storage pressure vessels. They continuously increased the pressure of the vessel, and after 324 MPa, suddenly the pressure fell to zero, indicating bursting. Zheng et al. [45] conducted a fatigue test to analyze the behavior and fatigue life of the carbon fiber/epoxy composite. They found that after 500 cycles, the composite faced fatigue bursting; at that moment, pressure could be significantly decreased by approximately 15%. Feng et al. [46] analyzed the welded joints of TC4 titanium alloys for storage tanks, considering 10,000, 20,000, and 30,000 pre-cycles. After 24 h of electrochemical hydrogen charge on the specimen, the failure process was studied using SEM and TEM. The results show that the initial dislocation density of the specimens rises with the number of pre-cycles, making the hydrogen embrittlement through aging more severe. Following 10,000 and 20,000 pre-cycles, the specimens exhibited fewer dislocations and hydrogen capture at specific sites. In contrast, after 30,000 pre-cycles, dislocation accumulation and entanglement appeared both along the phase boundary and within the phase, resulting in a greater number of hydrogen capture sites. At lower cycles, the HELP mechanism dominates the tensile process, whereas the HEDE mechanism dominates the process at higher cycles.

Yamabe et al. [35] conducted a laboratory test where a high-pressure O-ring seal was used as a specimen. The O-ring was made of low-nitrile NBR (acrylonitrile content: 18%), which was vulcanized by sulfur and filled with carbon black. With a 30% compression ratio, the specimen was compressed in a radical direction. Then, it was inserted into a vessel that was pressurized by hydrogen from the bottom. They conducted optical and scanning electron microscopy in post-aging experiments to observe the cracks. Additionally, they conducted gas chromatography–mass spectroscopy to measure the hydrogen gas content and a densimeter to measure the volume increase. They found two types of cracking for failure: one started from the center of the O-ring cross section and another from near the surface. Brownell et al. [47] conducted a simulation on ethylene-propylene-diene-based elastomeric rubber; they used O-rings and hose liners as specimens and exposed the sample to a pressurized hydrogen environment. Due to high pressure and rapid decompression, the specimen failed as a result of cavitation and stress-induced localization of hydrogen gas. This result supports the experimental result of the NBR O-ring.

Wilson et al. [48] conducted the same experiment as Brownell et al. [47]. They just increased the crosslinking of the polymer using sulfur and found crosslinks create extra free volume at a pressure that facilitates the localization tendency of the hydrogen gas. Zhou et al. [49] modified the rubber seal materials, including the O-ring and D-ring, and conducted a simulation using finite element analysis. Their results show that the O-rings have a higher tendency to fail under stress concentration compared to the D-rings under hydrogen pressure ranges of 0–100 MPa. At high hydrogen pressure and contact stress, the D-ring outperformed the O-ring in terms of sealing ability, but the inverse was true under low pressure. They also analyzed the friction effect on the failure and found the D-ring had better performance against friction compared to the O-ring at high pressure.

### 2.4. Mechanical–Chemical Aging

The mechanical aging of materials is a slow, progressive, and irreversible process of losing the capability to perform the assigned work under a high mechanical loading condition over time. Whereas chemical aging is a long-term interaction with the chemical environment, and due to this interaction, material’s structure, composition, and morphology change permanently. When a material is exposed to a high mechanical load and chemical environment simultaneously, the process is called mechanical–chemical aging. 

#### 2.4.1. Mechanism of Mechanical–Chemical Aging

**Figure 7:**
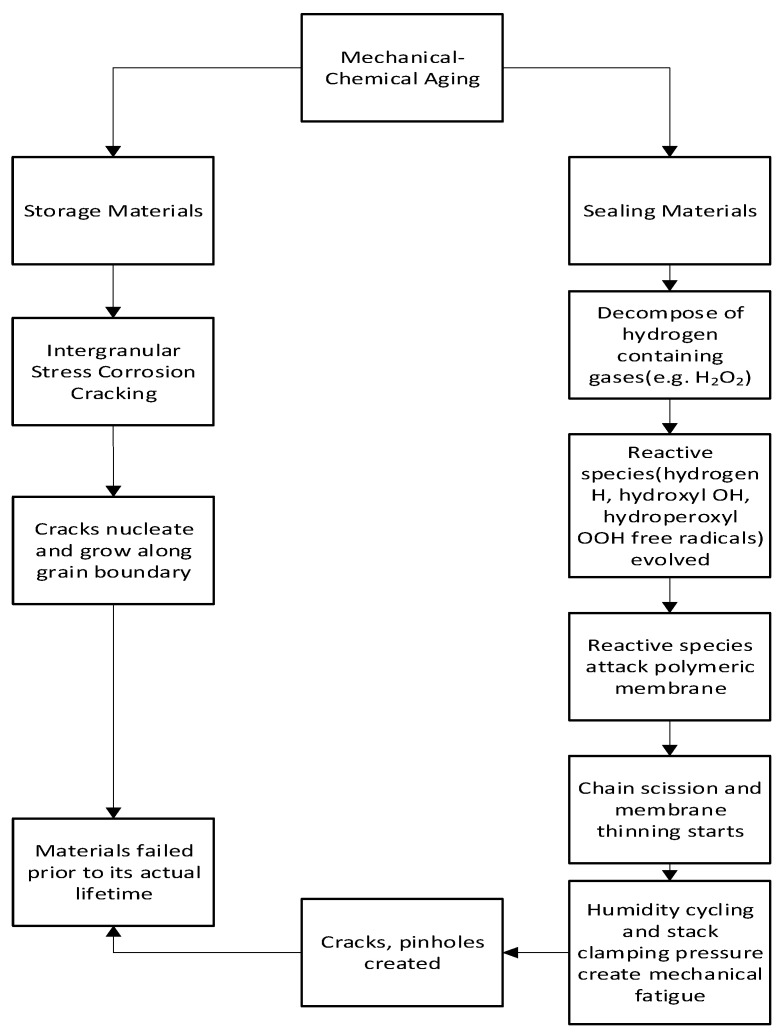
Flowchart of the mechanism of mechanical–chemical aging [50,51,52,53,54,55,56,57].

Hydrogen storage and sealing materials are subjected to mechanical–chemical aging during their normal operational cycle. Figure 7 shows the flowchart of mechanical–chemical aging. 

#### 2.4.2. The Laboratory Test for Mechanical–Chemical Aging

Harris et al. [58] conducted a laboratory test on Monel K-500, a precipitation-hardened Ni-Cu alloy susceptible to intergranular stress corrosion cracking under a hydrogen environment at 923 K. The specimen was immersed in a 0.6M NaCl solution. A saturated calomel electrode was used to apply a potential was −1000 to −1200 mV. In four heat treatment conditions, the under-aged and peak-aged specimens showed increasing susceptibility to intergranular stress corrosion cracking compared to an un-aged and over-aged specimen. Nykyforchyn et al. [59] conducted a laboratory test where they tested ferrite pearlitic X52 steel cylindrical, smooth, and pre-cracked samples into a solution of artificial brine mimicking onshore conditions where stress corrosion cracking could be created. The test was conducted both in the presence and absence of external polarization. In the absence of external polarization, aggressive environments did not show as much effect as in the presence of external polarization. In the presence of external polarization, both specimens were affected.

Robert et al. [60] took a perfluorosulfonic acid-based membrane and placed it in a degradation solution. At one time, the membrane was fitted with a universal testing machine for mechanical stress. Two types of solutions were used: one is mild (3 Vol% H_2_O_2_) and another was an aggressive solution called Fenton solution (3 Vol% H_2_O_2_ + 1 ppm Fe^2+^ ion). Mechanical fatigue was applied to create mechanical stress on the membrane. A cyclic compressive stress was applied on the channel ribs to reproduce one-hand swelling or shrinkage cycles, and a static compressive stress was maintained for the stack clamping pressure on the membrane. In this experiment, fluoride emission rates (FER) were observed to predict the decomposition of the polymeric membrane. The post-aging condition of the material was determined by Robert et al. [60] by using a fluoride-ion selective electrode, which measured the amount of emission of fluoride ions. It was considered a reliable PFSA (perfluorosulfonic acid) chemical degradation indicator. From the analysis of FER data, it was confirmed that aging was taking place there.

### 2.5. Thermo-Mechanical Aging

Combining both the thermal and mechanical aging processes, when high mechanical load and temperature work at the same time for the aging of material, it is called thermo-mechanical aging. Such conditions are generally created within hydrogen fuel cells and sealing materials.

#### 2.5.1. Mechanism of Thermo-Mechanical Aging

**Figure 8:**
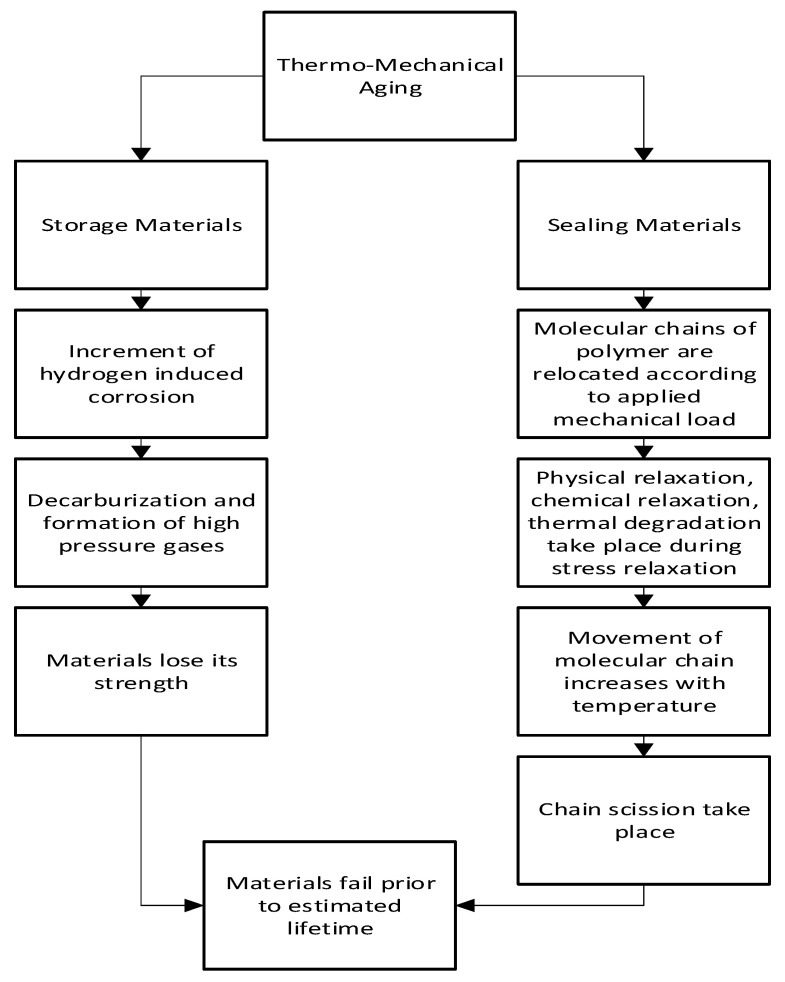
Flowchart of the mechanism of thermo-mechanical aging [17,61,62,63,64,65].

The diffusion of hydrogen affects the thermo-mechanical aging method. This is mainly seen in polymeric materials with viscoelastic behavior, if the strain is held constant viscoelastic materials show stress relaxation, and if stress is held constant, they show creep. Physical relaxation, chemical relaxation, and thermal degradation are some combined factors during stress relaxation in the polymer. Figure 8 shows the flowchart of thermo-mechanical aging.

Thermo-mechanical aging occurs when hydrogen-induced corrosion in storage materials increases with increasing mechanical load and elevated temperature. Corrosion-induced hydrogenation of the materials, in combination with working stresses, may lead to the emergence of nano and microscale bulk damage. Hydrogen evolution may occur with corrosion, particularly in cracks caused by the local acidity of the corrosion media [17]. During operation, materials experience in-bulk repeated damage due to decarburization and the creation of high-pressure hydrogen gases, causing them to lose their strength of materials [64] 

In addition, when sealing material is subjected to mechanical load in a hydrogen environment, molecular chains of polymer are relocated. This causes stress relaxation which directly reduces the sealing force. Physical relaxation occurs first among other factors, then comes chemical relaxation, and later thermal degradation. With increasing temperature and operation cycles, the movement of the molecular chains increases which causes chemical reactions and chain scission. In this way, due to the stress relaxation behavior, polymeric material starts to loosen its sealing force as well as the sealing capability [65].

#### 2.5.2. Laboratory Test for Thermo-Mechanical Aging

Balitskii et al. [61] conducted a laboratory test on two steel specimens, 05Kh12N23T3MR and 10Kh15N27T3B2MR, in a high-pressurized hydrogen environment and at elevated temperatures. A static tensional force was also applied to the temperature. At room temperature, the hydrogen action on both samples was minimal. However, at high temperatures, 10Kh15N27T3B2MR was more sensitive to hydrogen embrittlement than 05Kh12N23T3MR but less sensitive than 60Kh3G8N8V and 12Kh18AG18Sh steel. NYKYFORCHYN et al. [62] conducted a laboratory test on Cr-Mo-V steel at 450 °C with an applied tensile load. Their results show that the degradation of Cr-Mo-V steel is increased, and the fatigue crack growth resistance is decreased due to hydrogen. Cui et al. [62] conducted a laboratory test using a liquid silicon rubber (LSR) gasket as a specimen. The test was performed in two mediums: air and water. Stress relaxation equipment was used in the experiment. During the experiment, constant deformation was applied to the sample, and the temperature was raised to 100 to 120 °C. The test was performed at different strain rates, temperatures, and stress levels. They used time–temperature superposition theory to construct a master curve that could predict the service life of the specimen. They found that the medium also affected the aging of the species. They found that at 70 °C, LSR service life could be 5000 h at 60% of initial sealing stress. Below the 60% initial sealing stress, it was considered leakage.

## 3. Durability Analysis of Hydrogen Sealing and Storage Materials through Aging Tests

### 3.1. Durability Test Methods and Principles

**Figure 9:**
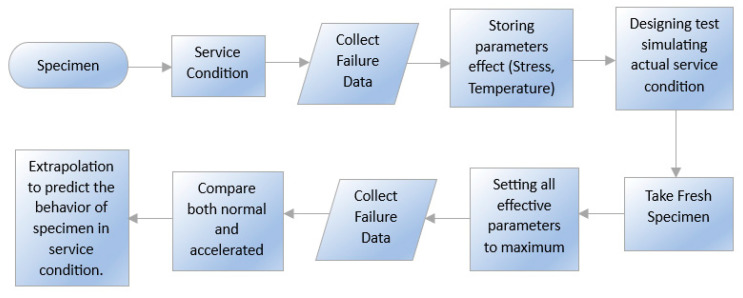
Process Diagram of Accelerated Durability Test [64].

An accelerated durability test (ADT) can be performed to predict the durability of materials. It can be performed to minimize the time and give a better prediction of durability. Under most operating conditions found in industry, material aging occurs over a long time. Materials tends to deteriorate rapidly if the regular conditions undergo a shift to severe ones. In ADT, this principle is followed. The test procedure is presented in Figure 9 with a flowchart. With this test, metallic hydrogen storage materials and polymeric sealing materials may be compared. This test is used not only for testing durability but also for material comparison, quality control, and design information. In this technique, specimens are compared under both normal and accelerated conditions. Chemical abrasiveness, temperature, and pressure are held at their highest levels to simulate the worst case scenario during an accelerated test cycle. Therefore, mechanical, chemical, electrochemical, and thermal failure mechanisms exist [66]. The specimens under normal and accelerated aging conditions are compared, and extrapolation is utilized to anticipate the service life and durability of the material [67,68].

The main limitation of this test is the simulation of service conditions, which is tough. As a result, the predicted value has errors within a specific limit. Again, the activity of the materials found in harsh conditions is not always reflected in mild conditions. The benefits of the ADT method are, this test is a comprehensive test for predicting the material’s durability, especially polymeric sealing materials [63]. Burgess et al. [64] conducted an accelerated durability test for a pressure relief device (PRD) in a hydrogen environment, where they created the worst conditions for the PRD that it could face in its service conditions. A medium-pressure hydrogen storage cylinder was used to conduct the test with the PRD under test acting as the only protective device Maximum stress and temperature cycles were applied to the specimen, and failure behavior was observed. From the results, they predicted the behavior of the 440C steel specimen. An accelerated stress test is a form of ADT used in the determination of the behavior of the proton-exchange membrane (PEM) fuel cell. Polymeric sealing materials can be compared with PEM. In this system, failure modes are mechanical, chemical, electro-chemical, and thermal. Thus, for an accelerated test stress cycle, chemical harshness, temperature, and pressure are kept at maximum to create the worst conditions for PEM. Liu et al. [65] and Kundu et al. [69] conducted accelerated stress test for the Nafion membrane. Liu et al. [65] conducted ADT for 10 cycles, and each cycle consists of 100 h. They found significant degradation taking place at 500–600 h of operation, and at 1000 h, the membrane was completely degraded. Kundu et al. [69] found that over 4–5 days of ADT, almost 20% of weight was lost. By extrapolating these data, the service life could be measured.

**Figure 10:**
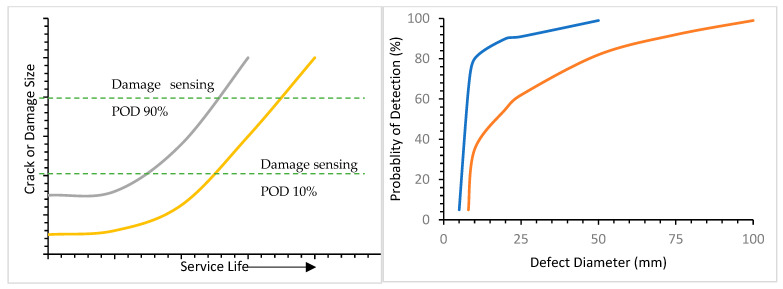
Graphical representation of service life with defect size and probability of detection (POD) [44].

The service life of a material can be defined as the amount of time a material can provide the proper service for which it was made. There is a relationship between service life and crack defect size. Figure 10 shows that after long-term use, when materials spent spend a significant portion of their service life, the distribution of cracks and their sizes increases. The service life of a material can be predicted using the reverse method. In this method, crack defect size is measured using ADT, and service life can be predicted from that value. For detecting cracks in materials in a hydrogen environment, several tests can be performed such as changes in the vibration characteristics of structure technique, acoustic emission technique, structure interrogation using lamb waves and ultrasonic using piezo transducer technique, eddy current, and ultrasonic technique [67]. Figure 10 assist to determine the distribution of equivalent initial flow size (EIFS) that can be calculated from the difference of two curves at initial point while the damage sensing (POD) is less than 10% and the distribution of the final lives can be obtained from the difference of two curves at end point while the POD is more than 90%. Distribution of detectable defect size can be measured from the difference of two curves at the mid-point where damage sensing is more than 10% and less than 90%.

The time–temperature superposition (TTS) principle is an established principle for predicting service life. It is a commonly used method where a master curve is created from data at selected reference temperatures. From this master curve, service life is predicted. The relationship between time (t) and temperature (T) for the mechanical response is described in the TTS principle [68,70] and the relation can be written mathematically as follows:

G_r_ (t, T_2_) = G_r_ (a_T_ (T_1_, T_2_) t, T_1_)

where G_r_ = mechanical response, a function of time and temperature; T_1_ and T_2_ = two different temperatures; t = time; a_T_ = shift factor.

**Figure 11:**
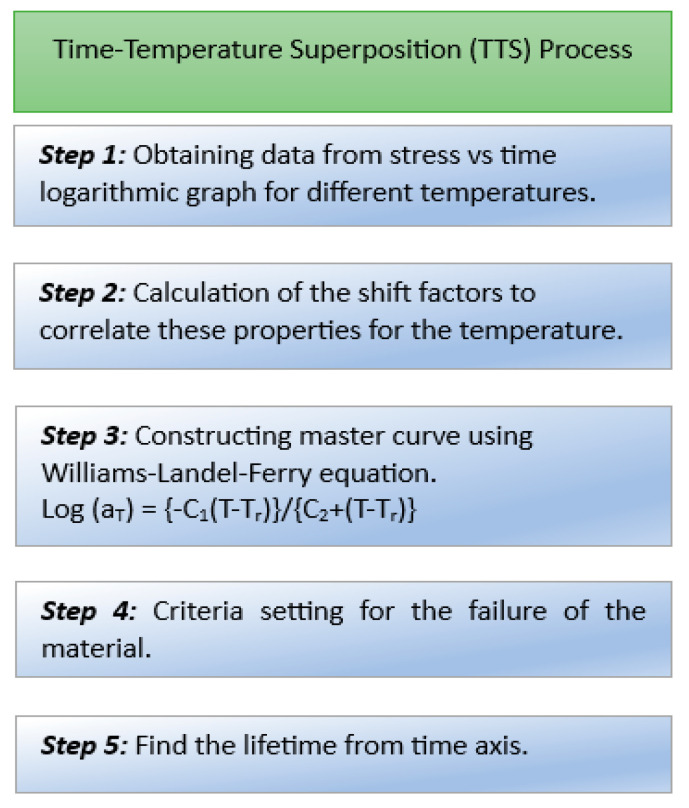
Process of predicting the lifetime of a material using the TTS process [66,72].

The principle states that time and temperature are equivalent. At two different temperatures, the same shape and time function of stress can exist. When the stress relaxation curve is presented on a logarithmic-logarithmic scale, this curve can be shifted to another temperature horizontally. The TTS principle is used to shorten the test time because it tests material for a shorter time at a higher temperature [62]. Creep behavior and the stress relaxation of polymeric materials can be determined using this principle [71]. For predicting the life of a material using TTS, the process shown in Figure 11 is followed.

Cui et al. [62] tested LSR at three different temperatures (70 °C, 100 °C, and 120 °C) and obtained a stress versus time logarithmic curve. Using the Williams–Landel–Ferry equation [72], they constructed a master curve and fixed 60% of stress for the threshold value of leakage. They then drew a line from 60% to the master curve. Then, another line was drawn from the master curve, intersecting at a point on the time axis. The value at which the line intersects the time axis is the predicted lifetime of the material.

### 3.2. Service Life of Storage and Sealing Material under Simultaneous Multiple Aging

In the case of a hydrogen environment, failure of the storage material can create a disaster. Taking the thermal aging of steel into consideration, O. Z. Student [27] found changes in the structure during laboratory aging for 40 h, which were observed after continuous operation of 140,000–190,000 h. Therefore, the service life of the steel can be predicted using ADT. Vessel chambers and transportation piping is susceptible to chemical interactions when used to store hydrogen. Under chemical aging conditions, no such work was found where anyone was claiming the service life of any storage materials. For thermal aging, the predicted service life was 140,000–190,000. However, in mechanical–chemical aging, the service life prediction was 5500 h, and in chemical aging, it was 6000 h, which is a huge difference [32,40]. Thus, the intensity of thermal aging is comparatively lower than other methods, and it also varies depending on the service condition, materials used, environment, etc.

In fuel cells, if the sealing material of the membrane ages and leaks, it can create a great disaster [73,74]. Taking the chemical aging of a polymeric gasket material into consideration, it was found that the test time under an ADT environment of 6 h could be equivalent to 27 h of the lifetime of the specimen. This result was based on the time-concentration superposition theory, and it showed that under harsh conditions, the extrapolating lifetime was four times the experimental time. By using the TTS principle based on stress relaxation behavior, it was predicted that the service life of Silicon rubbers would be at least 6000 h under the simulated fuel cell environment [75]. When mechanical and chemical aging occurs in a system, the condition worsens more rapidly. Because of chemical aging, the material becomes brittle and loses its elastic properties. If a mechanical load is applied to it at the same time, the process of aging will be accelerated as usual. A laboratory study found that under fuel cell operation where combined mechanical–chemical aging takes place, the service life of a membrane is approximately 5500 h [76].

One observable thing is that if the services are compared with each other, then the intensity of this aging can be understood. The service life of a membrane under chemical aging was predicted at 6000 h. However, when mechanical aging was added to the chemical aging, the service life was reduced to 5500 h. Thus, it is clear that the intensity of aging is higher in both mechanical and chemical aging. However, it is also important to know that chemical aging is more efficient than mechanical aging [60].

## 4. Key Factors Affecting Aging under the Hydrogen Environment

Temperature, aging time, stress, thermal cycle, environment, hydrogen pressure, and hydrogen distribution are the primary factors affecting aging in a hydrogen-rich environment. The literature indicates the impact of factors; Zelenak et al. [77] found that high temperatures elevate hydrogen aging, resulting in the formation of reactive metal hydride composites or complex metal hydrides in storage materials. Eliezer et al. [78] observed that the concentration of hydrogen had an impact on the aging behavior of titanium alloys in a hydrogen atmosphere, with greater concentrations resulting in more rapid aging., For an adequate assessment of the serviceability of materials in a hydrogen environment, it is essential to take into account the operating conditions that can influence aging behavior.

### 4.1. Effects of Temperature

**Figure 12:**
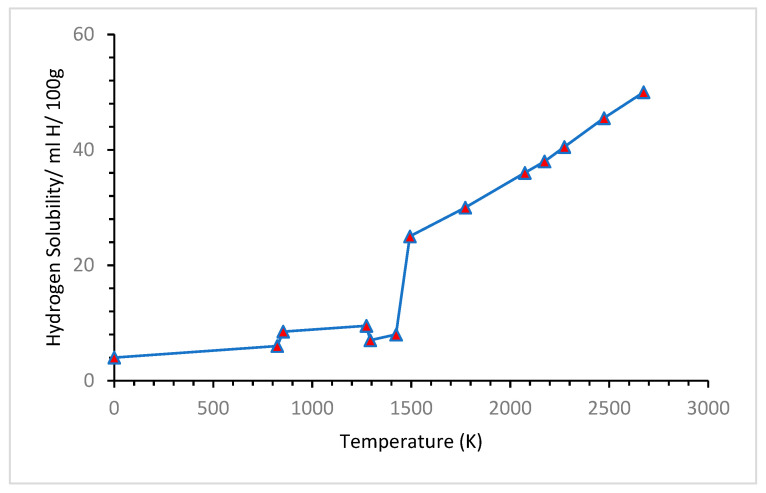
Hydrogen solubility vs. temperature curve [79].

The temperature effect on hydrogen storage metallic materials can be described from the metal–hydrogen interaction. Metals lose strength when hydrogen embrittlement occurs. This phenomenon depends on hydrogen absorption, physical adsorption, transportation of hydrogen to the crack tip, transportation of hydrogen to tensile stress regions, etc. All these factors are highly temperature dependent. Figure 12 shows how the solubility of hydrogen increases with temperature. It can be observed that the hydrogen solubility in molten iron above 773.15 K is approximately 30 ppm, and at 1273.15 K, the hydrogen solubility of molten iron is less than 10 mL H/100 g. It reached more than 40 mL H/100 g at a temperature above 2273.15 K [79].

**Figure 13:**
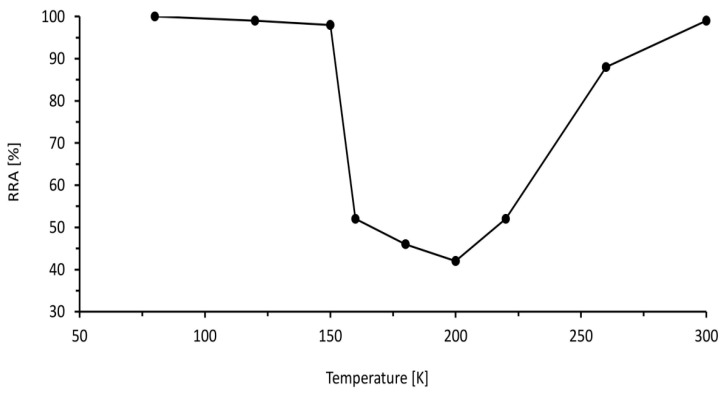
Relative reduction in the area of 316 Stainless Steel with the temperature at tensile test in a hydrogen environment [80].

Figure 13 describes the relative reduction in the area, which is a measure of ductility that varies with temperature. At lower temperatures from 75 K to 150 K, the effect of hydrogen on ductility is low due to the slow transportation rate, and at higher temperatures more than 250 K, the hydrogen effect is low as well because of the trapping of hydrogen by dislocation. However, in the intermediate region from 150 K to 250 K, hydrogen has a significant impact on the ductility of the metal. In this intermediate region, the ductility of metal reduces significantly to approximately 40%. 

At cryogenic temperature for storing liquid hydrogen, materials face embrittlement. It is known that depending on crystal structure the embrittlement tendency varies. Face centered cubic crystal structure has lower embrittlement tendency than body centered cubic crystal structure. This is because body centered cubic structures have lower activated slip system at low temperature. As a result, face centered cubic structured materials have high sustainability in cryogenic temperature. Ductile to brittle transition temperature is another important parameter which needs to be consider. The materials that have ductile to brittle transition at low temperature can be significantly affected if used for low temperature application [81]. 

The effect of temperature due to hydrogen differs from material to material. For polymeric gasket materials, Wu et al. [82] found that with increasing temperature, the mechanical properties of the sealing materials, including the tensile strength, elongation at failure, compression stress relaxation, and compression permanent deformation, tend to decay. Dubovsk et al. found [83] in their work that high temperatures accelerate the degradation of the seal. Stress relaxation of polymeric sealing materials is dependent on the temperature. Chemical relaxation and thermal degradation increase with increasing temperatures [62]. For polymeric materials the, with decreasing temperature bonding strength between molecular chains increases which increases the Young’s modulus as well as the tensile strength but the toughness decreases [84,85]. 

**Figure 14:**
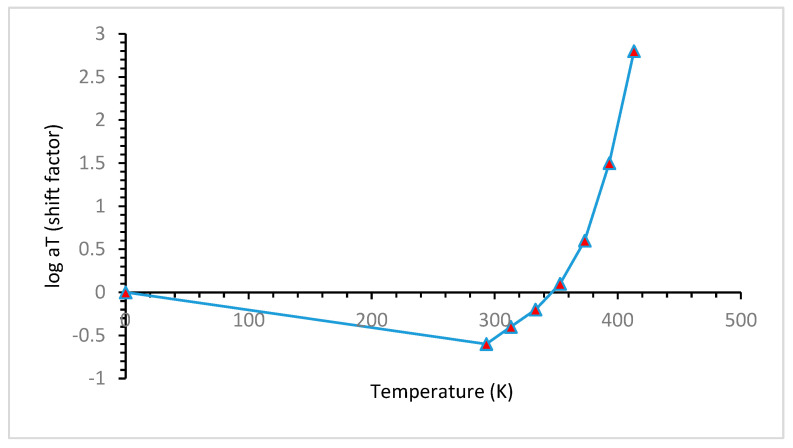
Shift factor of the master curve [62].

Figure 14, indicates that temperature has a considerable effect on the stress relaxation of polymeric materials, and with increasing temperature shift factor increases sharply. The shift factor is the degree of material’s time acceleration in an isothermal environment. As the shift factor increases with temperature, the materials’ aging accelerated.

### 4.2. Effect of Pressure

Hydrogen permeation into the metal increases with increasing pressure, which enhances the embrittlement tendency of the metal. When hydrogen is stored in a container, it exerts pressure on the container wall. If the container becomes metal, the permeation of hydrogen through the metal can be higher, and the metal’s aging tendency can increase [86]. The increasing pressure also increases the interaction between the hydrogen and the sealing materials. As a result, the absorption of hydrogen increases, which eventually degrades the properties of the sealing material, as discussed earlier [87]. Another important phenomenon takes place when high pressure is suddenly released. Due to the sudden drop in pressure, the volume of the inside gas increases, and desorption starts. During the desorption process, bubbles are created, which create microcracks; this phenomenon is known as cavitation [48].

**Figure 15:**
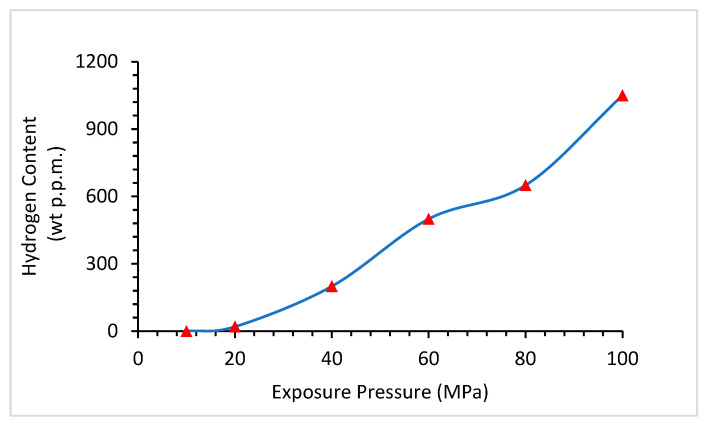
Relationship between Hydrogen Content and Exposure Pressure [86].

Figure 15 shows that with increasing pressure, hydrogen has a higher tendency to dissolve in materials. At 20 MPa, the hydrogen content was less than 50 ppm, which reached almost 1000 ppm at 100 MPa. Because hydrogen is very small in size and increases in pressure, it can easily penetrate through materials.

### 4.3. Effects of Stress

Stress is an important factor that can significantly affect the aging process in a hydrogen environment. There are different types of stresses, such as thermal stress, chemical stress, and mechanical stress. Thermal stress is induced in materials due to temperature fluctuations. It can facilitate the aging process, and this type of stress is highly related to thermal cycling. In harsher chemical conditions, chemical stress increases.

**Figure 16:**
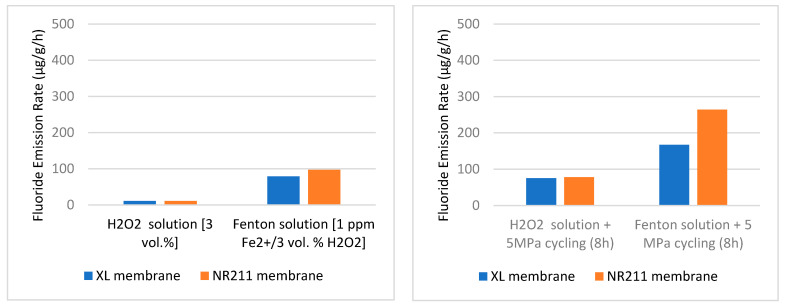
The fluoride emission rate under different stress conditions (static, cyclic) [62].

Robert et al. [62] conducted an aging test using a perfluoro-sulfonic acid-based membrane. Two membranes were selected for the experiment, and both were Nafion based membrane, Nafion™ NR211 and Nafion™ XL. The XL membrane differs from the NR211 by the presence of additional PTFE-rich reinforcement and cerium-based radical scavengers. This type of membrane is degraded by the emission of fluoride ions when it reacts with chemicals. They tested their specimen in two stress conditions, static and cyclic. After testing, they found that when cyclic stress was introduced instead of static stress, the fluoride emission rate of the specimen was increased. Figure 16 shows the results of their experiments. The first graph indicates static stress condition and the second graph is cyclic stress condition The results indicate the fluoride emission rate is higher in cyclic conditions and this is indicative of an aging process having occurred. 

### 4.4. Effects of Thermal Cycle

**Figure 17:**
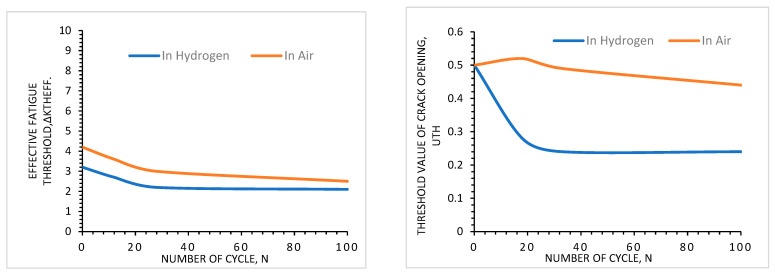
Threshold values for fatigue failure of materials in air and hydrogen [27].

Thermal cycling is a cyclic process of rapid temperature change where a material remains at a higher extreme in one phase and a lower extreme in the other. Due to thermal cycling, thermal stress is created in materials, which is called thermal aging. Thermal aging results from fatigue crack growth and depends on the number of cycles and material properties. Not only does the number of cycles affect the aging process, but the range of the temperature of the thermal cycle is also significant. A higher range of temperature differences can cause more variation in the diffusion of hydrogen, which can affect the aging process [27]. Figure 17 shows that an increase in the number of thermal cycles increases the tendency for fatigue failure of materials. In the left-side figure, the threshold value for effective fatigue decreases with increasing the number of cycles. Because it is known that fatigue is a cyclic stress-dependent failure mechanism that increases with increasing stress cycles, the important thing to notice here is the behavior of hydrogen. When the test is performed in a hydrogen environment instead of air, the threshold value decreases more than in air. This evidence proves that thermal cycling affects the aging mechanism of materials in a hydrogen environment. The right-side figure also describes the same type of effect, where the threshold value for crack opening decreases with an increasing number of cycles and hydrogen environment.

### 4.5. Effects of Environment

**Figure 18:**
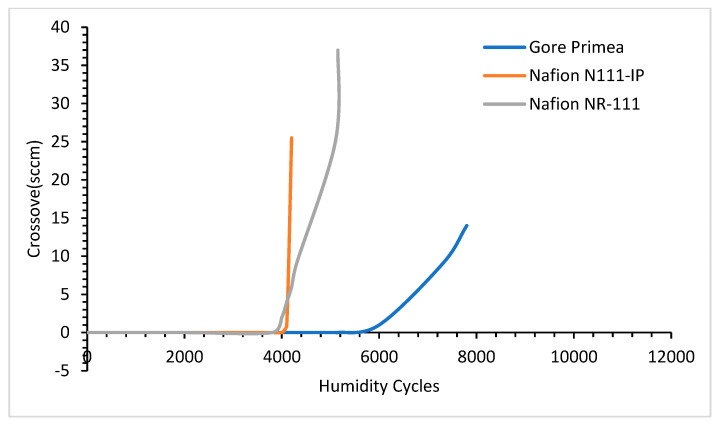
Crossover leakage tendency of different types of materials in different humidity cycles [76].

The environmental effect has a impact on the aging of sealing and storage materials. These materials work in extreme conditions where they may be affected by the humidity cycle, harsh chemical environments such as a strongly acidic environment. As the humidity increases, the crossover leakage tendency increases. The crossover leakage is the tendency of the mixing of different gases (such as in a proton-exchange membrane fuel cell H_2_ and O_2_) leaking through the gasket material. Gittleman et al. [76] tested three types of membranes, Gore Primea, Nafion N111-IP, and Nafion NR-111, in different humidity cycles. The result of the crossover tendency is shown in Figure 18. Figure 18 shows that by increasing the humidity cycle to the threshold value crossover tendency of every material increases in a hydrogen environment. This evidence is indicative of that the humidity cycle influences the hydrogen aging of the materials. 

A harsher environment also affects the hydrogen aging process of the materials. In Figure 16, the H_2_O_2_ environment is mild, whereas the Fenton solution environment is harsh. The graph shows when the environment approaches harsh conditions the fluoride emission rate increases significantly, which means the aging is also increasing. The ambient pressure of hydrogen also affects the aging process. Cracks are formed in materials due to the fluctuation of the hydrogen pressure. This pressure creates stress on the defective sites and forms cracks [27]. 

### 4.6. Effects of Alloying Elements

Alloying elements have effects on the degradation of hydrogen storage metallic materials. Martin et al. [88] found that increasing alloying elements percentage in stainless steel increases the relative reduction in area in the hydrogen environment.

**Figure 19:**
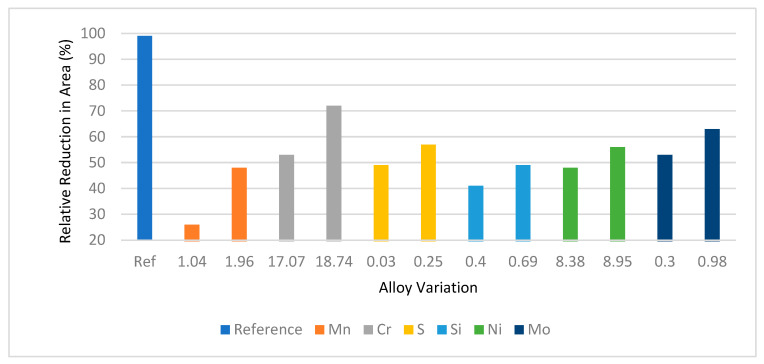
Relative reduction in area depending on different alloying elements [88].

Figure 19 shows that the hydrogen embrittlement tendency of stainless steel decreases with increasing alloying elements. They used Mo, Ni, Si, S, Cr, and Mn as alloying elements. Among these alloying elements Si, Mn, and Mo show much resistance to hydrogen embrittlement compared to others. 

In high-strength steel, chemical heterogeneity is intentionally created by introducing manganese (Mn), enhancing hydrogen resistance. Sun et al. [89] found manganese (Mn) in high-strength steel created a manganese-rich zone within the microstructure which restricted the stability of the hydrogen phase and arrested the microcracks produced by hydrogen. In aluminum alloys, alloying elements have two different types of effects. Anyalebechi [90] found adding Cu, Si, Zn, and Fe decreases hydrogen solubility, whereas Mg, Li, and Ti increase solubility. Therefore, some alloying elements have the tendency to increase hydrogen solubility in the alloy and some others have the tendency to restrict it. The alloying elements which increase the hydrogen solubility tendency in alloys are responsible for increasing the aging tendency of material in a hydrogen environment. 

## 5. Research Gap Analysis

It is crucial to predict the operational degradation of laboratory materials, especially for novel materials, manufacturing, or processing technologies. There is not enough research on the stability of the complex properties of the materials for long-term operations in a hydrogen environment. A comprehensive exploitation should be considered prior to proposing any operation, such as a new pipe welding technology with higher performance, higher mechanical properties, and high resistance to hydrogen-induced cracking. Welding is a common fabrication method due to hydrogen-induced cracking during welding, accelerated aging occurs and materials fail before their full expected lifetime. For most metal alloys, no known published data are found for creep, fatigue, or the impact of gaseous hydrogen on storage materials [86], which indicates limited general knowledge about the materials and their performance in the hydrogen environment. There is a gap in research on material grades, industry-mandated parameters, and operating conditions [3]. Therefore, hydrogen compatibility with the mechanical properties of storage materials needs to be extensively studied.

According to Ogawa et al., the precipitation-hardened alloy shows low HE susceptibility and high thermal conductivity on the lab scale [33]. The fracture toughness may be further increased by adding the required alloying elements and appropriate heat treatments to enable practical applications such as heat exchangers exposed to high-pressure hydrogen. Given that, several types of hydrogen damage have been observed in pipeline steel with various microstructures, the impact of each microstructural phase on HE needs to be elucidated through careful investigation. Some researchers contend that martensitic microstructure or ferritic pearlite are less preferred for HIC resistance than bainitic ferrite and acicular ferrite; however, the available data have remained either conflicting or less convincing [18,91,92]. The impact of hydrogen vacancy interactions on ductility, quasi-brittleness, and fatigue has yet to be explicitly explored. Srinivasan et al. reported that hydrogen vacancy interaction has a detrimental impact on austenitic and ferrite steels as well as Ni alloys, but further investigation is necessary. It has been observed that hydride formation plays an important role in the hydrogen embrittlement behavior of alloys [93]. However, the hydrogen charging conditions under which hydrogen formation would be negligible have not yet been thoroughly investigated. According to Michler et al. [94] the hydrogen embrittlement effect for metals in a hydrogen environment normally begins at the gas–metal interface. Mechanisms related to hydrogen–material interactions are likely the rate-limiting steps in the hydrogen reaction chain [94]. There is a lack of understanding about the mechanisms that would allow such effects to be measured. Simulation tools may offer a framework to investigate, given that calculations using density functional theory have shown positive results.

According to the literature, it is asserted that the experimental hours correspond exactly to the amount of in-service degradation. For instance, it is stated that 140,000 to 190,000 h of in-service degradation are equivalent to a lab experiment lasting 40 h [27]. However, the correlation between service life and laboratory accelerated degradation is not explicitly stated, as laboratory experiments are often conducted in a controlled environment, and l working conditions differ when a material operates in a hydrogen environment. There is still uncertainty regarding the precise mechanism by which hydrogen damage can occur in non-hydride-forming materials. Multiple mechanisms have been proposed, such as adsorption-induced dislocation emission (AIDE), hydrogen-enhanced decohesion (HEDE), and hydrogen-enhanced localized plasticity (HELP). These can take place simultaneously with stress, temperature, and the environment [18]. The ideal failure mechanism for HE is still not understood due to the difficulties in obtaining data at the atomic level and the inadequate capabilities of characterizations.

The literature discussed hydrogen aging by imbibing it in different chemical solutions, such as H_2_O_2_ or acidic fluoride solutions, or by combining it with natural gas rather than pure hydrogen. Liquid or gaseous hydrogen is directly used in the storage or pipelines of hydrogen infrastructure and the results collected in the lab may differ to varying degrees. Pipelines made of high-strength steel are susceptible to higher fatigue crack growth (FCG) rates in a hydrogen environment. Lower loading rates and load cycle frequencies, as well as load dynamics, can also affect FCG rates and be subjected to changes at different process conditions or up-sets. Existing models and simulations have been developed assuming minor displacement conditions and microscale yielding, which are typically not true for high-strength pipeline steels; hence, scaling them up to greater scales is challenging.

Without applying external stress, the hydrogen concentration at the interface is considered minimal, which results in a lower alloy embrittlement effect. However, the interaction of hydrogen at the interface is a complicated phenomenon that is not yet explicitly understood. Extensive research on simulating hydrogen diffusion with the involvement of numerous bulk cracks is still lacking. It is challenging to predict structural integrity when several cracks are present in a hydrogen atmosphere. To better comprehend and explain the propagation of such cracks due to strong field interactions at the tip of the crack, several speculative models have been put forth in the literature. However, the distribution and amount of absorbed hydrogen cannot be well described by any model, preventing it from providing sufficient details.

In chemical aging, the absorption of hydrogen increases the hardness of metals and leads to hydrogen embrittlement. However, the impact of hydrogen on polymeric materials has not yet been comprehensively investigated. Sealing materials exposed to high-pressure hydrogen cycles and high decompression rates may suffer internal damage, such as cavitation. Therefore, the wear and friction characteristics of sealing materials used in the hydrogen infrastructure must be studied to comprehend their compatibility in a high-pressure hydrogen atmosphere.

Raising the crosslink density of O-ring materials can lessen volumetric expansion upon decompression and lead to reduced free-volume pore dimensions [48]. As a result, identifying possible sites for hydrogen gas localization is the first step toward cavitation-induced failure. However, the damage progress can only be assessed for transparent graded O-rings, and only 2D projections of cavities can be provided [95]. Improved barrier properties were seen when Sun et al. and Bandyopadhyay et al. used 2D filler approaches in rubber materials to reduce hydrogen gas permeability [96,97]. However, there is significant room for research into adding novel filler approaches to minimize the current limitations of polymeric seals in hydrogen energy systems.

## 6. Conclusions

Sustainable energy sources and with eco-friendly behavior are two of the most important global demands of today’s world. Hydrogen, one of the most abundant elements in the universe, has the ability to fulfill this demand. However, storing and sealing hydrogen are very challenging due to its high aging tendency. Materials in a hydrogen environment degrade and deteriorate faster than expected and fail before their expected lifetimes. This review article discusses different types of aging that materials face within a hydrogen environment, such as thermal aging, chemical aging, mechanical aging, mechanical–chemical aging, and thermo-mechanical aging, along with their mechanisms. Some laboratory-based aging tests are also discussed as an examples of these aging processes. Different types of parameters that can affect the aging process are also discussed such as temperature, pressure, stress, thermal cycle, environment, and other alloying elements. For predicting the service life of materials, time–temperature superposition theory is a very popular method. The accelerated durability test is a common aging test that helps time–temperature superposition theory predict service life. The principles of both methods are discussed in this paper. At the end of this review article, different research gaps are discussed where further research is needed for effective storage and sealing of hydrogen. It is essential to store and seal hydrogen efficiently. Because the future of the hydrogen industry is highly dependent on these, without effective storage and sealing, the application of this energy source can be significantly hampered, as discussed in this article.

## Figures and Tables

**Table 1:** The energy content in hydrogen and other major fuels by weight and volume [4].

	Hydrogen	Natural Gas	Petrol
Energy content per unit mass	120.4 MJ/kg	51.6 MJ/kg	43 MJ/kg

**Table 2:** Different incidents that resulted from aging.

Incident	Location and Casualties	Cause	Description	Reference
The Flixborough disaster	England, 1974 28 killed, 36 injured	Cyclohexane plant leakage and gas cloud explosion	The chemical plant had a cyclohexane plant, where the reactor circuit leaked due to aging. As a result, a huge hydrocarbon gas cloud formed, which led to an explosion.	[11]
The Humber Refinery explosion	England, 2001 2 injured	Pipe corrosion and rupture due to fouling problems and blockages from accumulated water	Steel pipe failure was the main reason behind the explosion. The steel pipe was coated with a passive layer of iron sulfide which was protecting it from corrosion. However, due to the aging, the protecting layer ruptured, and the pipe surface underwent corrosion. As a result, the thickness of the pipe was reduced to 0.3 mm from 7–8 mm and could not contain the internal pressure, causing it to burst.	[12]
The Chevron refinery accident	USA, 2012	Pipe rupture by aging due to sulfidation corrosion	The main reason for this accident was the wall thinning of the pipe due to aging, which led to a catastrophic pipe rupture. Sulfur compounds found in crude oil feed, such as hydrogen sulfide, react with steel piping and equipment to cause sulfidation corrosion.	[13]
Muskingum Power Plant	USA, 2007	Storage tank failure and hydrogen explosion	The disaster began with the premature failure of the ruptured disk on the storage tank because of aging. The pressurized gas then caused the vent system to fail, allowing hydrogen to escape and causing an explosion.	[14]
Tesoro refinery	USA, 2010	Heat exchanger damage and tubing rupture	The accident was the result of damage to the heat exchanger brought on by hydrogen aging at high temperatures, which led to carbon steel tubing being badly fractured and weakened, which in turn led to a rupture.	[15]
Kashima Oil Refinery	Japan, 1982 5 casualties	High-pressure hydrogen aging and pipe deterioration	The explosion was caused by hydrogen aging under high pressure and high pressure in a hydrodesulfurization unit. Five casualties were caused by the gradual deterioration of the inner surface of a carbon steel pipe, which resulted in the discharge of process fluid after 12 years of operation.	[16]

## Data Availability

Data are contained within this article.

## References

[1] Yue M., Lambert H., Pahon E., Roche R., Jemei S., Hissel D. (2021). Hydrogen energy systems: A critical review of technologies, applications, trends and challenges. Renew. Sustain. Energy Rev..

[2] Nicoletti G., Arcuri N., Nicoletti G., Bruno R. (2015). A technical and environmental comparison between hydrogen and some fossil fuels. Energy Convers. Manag..

[3] Balasooriya W., Clute C., Schrittesser B., Pinter G. (2022). A review on applicability, limitations, and improvements of polymeric materials in high-pressure hydrogen gas atmospheres. Taylor Fr..

[4] Fassbender L. (2010). Hydrogen Safety Training for First Responders. https://www.hydrogen.energy.gov/docs/hydrogenprogramlibraries/pdfs/review10/scs015_fassbender_2010_o_web.pdf.

[5] Li H., Cao X., Liu Y., Shao Y., Nan Z., Teng L., Peng W., Bian J. (2022). Safety of Hydrogen Storage and Transportation: An Overview on Mechanisms, Techniques, and Challenges. Energy Rep..

[6] Barrera O., Bombac D., Chen Y., Daff T.D., Galindo-Nava E., Gong P., Haley D., Horton R., Katzarov I., Kermode J.R. (2018). Understanding and mitigating hydrogen embrittlement of steels: A review of experimental, modelling and design progress from atomistic to continuum. J. Mater. Sci..

[7] Eghbali P., Gürbüz M.U., Ertürk A.S., Metin Ö. (2020). In situ synthesis of dendrimer-encapsulated palladium(0) nanoparticles as catalysts for hydrogen production from the methanolysis of ammonia borane. Int. J. Hydrogen Energy.

[8] Handbook on Ageing Management for Nuclear Power Plants—Google Scholar. https://scholar.google.com/scholar?hl=en&as_sdt=0%2C5&q=Handbook+on+Ageing+Management+for+Nuclear+Power+Plants&btnG=.

[9] Hansler R.J., Bellamy L.J., Akkermans H.A. (2022). Ageing assets at major hazard chemical sites—The Dutch experience. Saf. Sci..

[10] Research Report 509—Plant ageing: Management of…—Google Scholar. https://scholar.google.com/scholar?hl=en&as_sdt=0%2C5&q=Research+Report+509+-+Plant+ageing%3A+Management+of+equipment+containing+hazardous+fluids+or+pressure+%282006%29&btnG=.

[11] ARIA (2008). Catastrophic Explosion of a Cyclohexane Cloud: Flixborough United Kingdom. http://www.aria.developpement-durable.gouv.fr/wp-content/files_mf/FD_5611_flixborough_1974_ang.pdf.

[12] Health and Safety Executive Public Report of the Fire and Explosion at the ConocoPhillips Humber Refinery on 16 April 2001. Health and Safety Executive. https://www.yumpu.com/en/document/view/22334776/public-report-of-the-fire-and-explosion-at-the-conocophillips-hse.

[13] U.S. Chemical Safety Board (2015). Chevron Richmond Refinery Pipe Rupture and Fire. Report No. 2012-03-I-CA-26. https://www.csb.gov/chevron-richmond-refinery-fire/.

[14] Case Study: Power Plant Hydrogen Explosion—WHA International, Inc. https://wha-international.com/case-study-power-plant-hydrogen-explosion/.

[15] CSB Investigation Finds 2010 Tesoro Refinery Fatal Explosion Resulted from High Temperature Hydrogen Attack Damage to Heat Exchanger—General News—News|CSB. https://www.csb.gov/csb-investigation-finds-2010-tesoro-refinery-fatal-explosion-resulted-from-high-temperature-hydrogen-attack-damage-to-heat-exchanger/.

[16] Diebold U. (2003). The surface science of titanium dioxide. Surf. Sci. Rep..

[17] Slobodyan Z.V., Nykyforchyn H.M., Petrushchak O.I. (2002). Corrosion resistance of pipe steel in oil-water media. Mater. Sci..

[18] Ohaeri E., Eduok U., Szpunar J. (2018). Hydrogen related degradation in pipeline steel: A review. Int. J. Hydrogen Energy.

[19] Sun Y., Cheng Y.F. (2021). Thermodynamics of spontaneous dissociation and dissociative adsorption of hydrogen molecules and hydrogen atom adsorption and absorption on steel under pipelining conditions. Int. J. Hydrogen Energy.

[20] Hayden L.E., Stalheim D. (2010). ASME B31.12 Hydrogen Piping and Pipeline Code Design Rules and Their Interaction with Pipeline Materials Concerns, Issues and Research. Am. Soc. Mech. Eng. Press. Vessel. Pip. Div. PVP.

[21] Sharma S., Ghoshal S.K. (2015). Hydrogen the Future Transportation Fuel: From Production to Applications. Renew. Sustain. Energy Rev..

[22] Yamabe J., Nishimura S., Koga A. (2009). A Study on Sealing Behavior of Rubber O-Ring in High Pressure Hydrogen Gas. SAE Int. J. Mater. Manuf..

[23] Zubrin R. (2007). The Hydrogen Hoax. New Atlantis.

[24] Nykyforchyn H., Tsyrulnyk O., Zvirko O., Hredil M. (2020). Role of hydrogen in operational degradation of pipeline steel. Procedia Struct. Integr..

[25] Tiwari G.P., Bose A., Chakravartty J., Wadekar S., Totlani M., Arya, Fotedar R. (2020). A study of internal hydrogen embrittlement of steels. Mater. Sci. Eng. A.

[26] Dwivedi S., Vishwakarma M. (2018). Hydrogen embrittlement in different materials: A review. Int. J. Hydrogen Energy.

[27] Student O.Z. (1998). Accelerated method for hydrogen degradation of structural steel. Mater. Sci..

[28] Shapovalov V.I. (1982). Influence of Hydrogen on the Structure and Properties of Iron-Carbon Alloys.

[29] Pokhmurskyi V.I., Fedorov V.V. (1998). Influence of Hydrogen on the Diffusion Processes in Metals.

[30] Krutasova E.I. (1981). Reliability of the Metal of Power-Generating Equipment.

[31] Nykyforchyn B., Andrusiv B.M., Voldemarov A.V., Kutsyn M.A. (1982). Evaluation of the effect of closure of fatigue cracks. Fiz.-Khim. Mekh. Mater..

[32] Hirakami D., Manabe T., Ushioda K., Noguchi K., Takai K., Hata Y., Hata S., Nakashima H. (2016). Effect of Aging Treatment on Hydrogen Embrittlement of Drawn Pearlitic Steel Wire. ISIJ Int..

[33] Ogawa Y., Yamabe J., Matsunaga H., Matsuoka S. (2017). Material performance of age-hardened beryllium–copper alloy, CDA-C17200, in a high-pressure, gaseous hydrogen environment. Int. J. Hydrogen Energy.

[34] Yamabe J., Takagoshi D., Matsunaga H., Matsuoka S., Ishikawa T., Ichigi T. (2016). High-strength copper-based alloy with excellent resistance to hydrogen embrittlement. Int. J. Hydrogen Energy.

[35] Yamabe J., Fujiwara H., Nishimura S. (2009). Fracture analysis of rubber sealing meterial for high pressure hydrogen vessel. Nihon Kikai Gakkai Ronbunshu A Hen/Trans. Jpn. Soc. Mech. Eng. Part A.

[36] Yokoyama K., Ogawa T., Takashima K., Asaoka K., Sakai J. (2007). Hydrogen embrittlement of Ni-Ti superelastic alloy aged at room temperature after hydrogen charging. Mater. Sci. Eng. A.

[37] Ogawa T., Oda T., Maruoka K., Sakai J. (2015). Effect of aging at room temperature on hydrogen embrittlement behavior of Ni-Ti superelastic alloy immersed in acidic fluoride solution. Int. J. Mech. Mater. Eng..

[38] Tal-Gutelmacher E., Eliezer D. (2005). Hydrogen cracking in titanium-based alloys. J. Alloy. Compd..

[39] Omura T., Nakamura J., Hirata H., Jotoku K., Ueyama M., Osuki T., Terunuma M. (2016). Effect of Surface Hydrogen Concentration on Hydrogen Embrittlement Properties of Stainless Steels and Ni Based Alloys. ISIJ Int..

[40] Li G., Zhu D., Jia W., Zhang F. (2021). Analysis of the aging mechanism and life evaluation of elastomers in simulated proton exchange membrane fuel cell environments. e-Polymers.

[41] Zhang E., Liu J., Zhang C., Zheng P., Nakanishi Y., Wu T. (2023). State-of-Art Review on Chemical Indicators for Monitoring the Aging Status of Oil-Immersed Transformer Paper Insulation. Energies.

[42] Wetegrove M., Duarte M.J., Taube K., Rohloff M., Gopalan H., Scheu C., Dehm G., Kruth A. (2023). Preventing Hydrogen Embrittlement: The Role of Barrier Coatings for the Hydrogen Economy. Hydrogen.

[43] Zhang M., Lv H., Kang H., Zhou W., Zhang C. (2019). A Literature Review of Failure Prediction and Analysis Methods for Composite High-Pressure Hydrogen Storage Tanks. Int. J. Hydrogen Energy.

[44] Klopffer M., Berne P., Castagnet S., Weber M. (2010). Pipes for Distributing Mixtures of Hydrogen and Natural Gas. Evolution of Their Transport and Mechanical Properties after an Ageing under an Hydrogen Environment. https://www.osti.gov/etdeweb/biblio/21400887.

[45] Zheng C.X., Wang L., Li R., Wei Z.X., Zhou W.W. (2013). Fatigue test of carbon epoxy composite high pressure hydrogen storage vessel under hydrogen environment. J. Zhejiang Univ. Sci. A.

[46] Feng X., Shi Y., Zhang W., Volodymyr K. (2023). Hydrogen Embrittlement Failure Behavior of Fatigue-Damaged Welded TC4 Alloy Joints. Crystals.

[47] Brownell M., Frischknecht A.L., Wilson M.A. (2022). Subdiffusive High-Pressure Hydrogen Gas Dynamics in Elastomers. Macromolecules.

[48] Wilson M.A., Frischknecht A.L. (2022). High-pressure hydrogen decompression in sulfur crosslinked elastomers. Int. J. Hydrogen Energy.

[49] Zhou C., Chen G., Liu P., Guohua C.Z., Liu C.P. (2018). Finite element analysis of sealing performance of rubber D-ring seal in high-pressure hydrogen storage vessel. J. Fail. Anal. Prev..

[50] Zatoń M., Rozière J., Jones D.J. (2017). Current understanding of chemical degradation mechanisms of perfluorosulfonic acid membranes and their mitigation strategies: A review. Sustain. Energy Fuels.

[51] LaConti A., Hamdan M., McDonald R.C. (2003). Mechanisms of membrane degradation. Handbook of Fuel Cells.

[52] Danilczuk M., Corns F.D., Schlick S. (2009). Visualizing chemical reactions and crossover processes in a fuel cell inserted in the esr resonator: Detection by spin trapping of oxygen radicals, nafion-derived fragments, and hydrogen and deuterium atoms. J. Phys. Chem. B.

[53] Lee S.Y., Cho E., Lee J.H., Kim H.J., Lim T.H., Oh I.H., Won J. (2007). Effects of Purging on the Degradation of PEMFCs Operating with Repetitive On/Off Cycles. J. Electrochem. Soc..

[54] Healy J. (2005). Aspects of the chemical degradation of PFSA ionomers used in PEM fuel cellsx. Fuel Cells.

[55] Kusoglu A., Weber A.Z. (2014). A Mechanistic Model for Pinhole Growth in Fuel-Cell Membranes during Cyclic Loads. J. Electrochem. Soc..

[56] Gittleman C.S., Coms F.D., Lai Y.-H. (2012). Chapter 2—Membrane Durability: Physical and Chemical Degradation A2. Mod. Top. Polym. Electrolyte Fuel Cell Degrad..

[57] De Moor G. (2012). Understanding membrane failure in PEMFC: Comparison of diagnostic tools at different observation scales. Fuel Cells.

[58] Harris Z., Burns J.T. (2019). A The Effect of Isothermal Heat Treatment on Hydrogen Environment-Assisted Cracking Susceptibility in Monel K-500. Mater. Sci. Eng. A.

[59] Nykyforchyn H., Lunarska E., Tsyrulnyk O.T., Nikiforov K., Genarro M.E., Gabetta G. (2010). Environmentally assisted ‘in-bulk’ steel degradation of long term service gas trunkline. Eng. Fail. Anal..

[60] Robert M. (2021). The Impact of Chemical-Mechanical Ex Situ Aging on PFSA Membranes for Fuel Cells. Membranes.

[61] Balitskii A., Ivaskevych L. (2009). Temperature Dependences of Age-Hardening Austenitic Steels Mechanical Properties in Gaseous Hydrogen. https://www.researchgate.net/profile/Alexander-Balitskii/publication/281269596_Temperature_Dependences_of_Age-hardening_Austenitic_Steels_Mechanical_Properties_in_Gaseous_Hydrogen/links/55dd955908aeb41644aefe8e/Temperature-Dependences-of-Age-hardening-Austenitic-Steels-Mechanical-Properties-in-Gaseous-Hydrogen.pdf.

[62] Cui T., Lin C.W., Chien C.H., Chao Y.J., Van Zee J.W. (2011). Service life estimation of liquid silicone rubber seals in polymer electrolyte membrane fuel cell environment. J. Power Sources.

[63] Brown R.P. (1991). Survey of status of test methods for accelerated durability testing. Polym. Test..

[64] Burgess R., Post M., Buttner W., Rivkin C. (2017). High Pressure Hydrogen Pressure Relief Devices: Accelerated Life Testing and Application Best Practices. https://www.osti.gov/biblio/1408284.

[65] Liu D., Case S. (2006). Durability Study of Proton Exchange Membrane Fuel Cells under Dynamic Testing Conditions with Cyclic Current Profile. J. Power Sources.

[66] Fukushima K., Cai H., Nakada M., Miyano Y. (2009). Determination of Time-Temperature Shift Factor for Long-Term Life Prediction of Polymer Composites. https://iccm-central.org/Proceedings/ICCM17proceedings/Themes/Behaviour/AGINGMOIST&VISCOEPROP/INT-AGINGMOIST&VISCOEPROP/IF1.1Fukushima.pdf.

[67] Irving P.E. (2012). Development of service life prognosis systems for hydrogen energy devices. Gaseous Hydrog. Embrittlement Mater. Energy Technol. Mech. Model. Futur. Dev..

[68] Hiemenz P.C., Lodge T.P. (2007). Polymer Chemistry.

[69] Kundu S., Simon L., Fowler M.W. (2008). Comparison of two accelerated Nafion^TM^ degradation experiments. Polym. Degrad. Stab..

[70] Andrews R.D., Tobolsky A.V. (1951). Elastoviscous properties of polyisobutylene. IV. Relaxation time spectrum and calculation of bulk viscosity. J. Polym. Sci..

[71] Struik L.C.E. (1977). Physical aging in plastics and other glassy materials. Polym. Eng. Sci..

[72] Williams M.L., Landel R.F., Ferry J.D. (1955). The Temperature Dependence of Relaxation Mechanisms in Amorphous Polymers and Other Glass-forming Liquids. J. Am. Chem. Soc..

[73] Li G., Tan J., Gong J. (2012). Chemical aging of the silicone rubber in a simulated and three accelerated proton exchange membrane fuel cell environments. J. Power Sources.

[74] Tan J., Chao Y.J., Yang M., Lee W.K., Van Zee J.W. (2011). Chemical and mechanical stability of a Silicone gasket material exposed to PEM fuel cell environment. Int. J. Hydrogen Energy.

[75] Cui T., Lin C.W., Chien C.H., Chao Y.J., Van Zee J. (2011). Service life prediction of seal in PEM fuel cells. Conf. Proc. Soc. Exp. Mech. Ser..

[76] Gittleman C., Lai Y., Miller D. (2005). Durability of Perfluorosulfonic Acid Membranes for PEM Fuel Cells. https://www.researchgate.net/profile/Craig-Gittleman-2/publication/265075095_Durability_of_Perfluorosulfonic_Acid_Membranes_for_PEM_Fuel_Cells/links/547321ef0cf2d67fc035dff1/Durability-of-Perfluorosulfonic-Acid-Membranes-for-PEM-Fuel-Cells.pdf.

[77] Nák V.Z., Saldan I., Giannakoudakis D., Barczak M., Pasán J. (2021). Factors affecting hydrogen adsorption in metal–organic frameworks: A short review. Nanomaterials.

[78] Eliezer D., Böllinghaus T.H. (2012). Hydrogen effects in titanium alloys. Gaseous Hydrog. Embrittlement Mater. Energy Technol..

[79] Tomi T. (2015). Effects of Hydrogen upon the Properties of Thermo Mechanical Controlled Process (TMCP) Steel.

[80] Fukuyama S., Sun D., Zhang L., Wen M., Yokogawa K. (2003). Effect of temperature on hydrogen environment embrittlement of type 316 series austenitic stainless steels at low temperatures. J. Jpn. Inst. Met..

[81] Duthil P. Material properties at low temperature. Proceedings of the CAS-CERN Accelerator School: Superconductivity for Accelerators.

[82] Wu Y., Wang D., Zhang W., Zhang J. (2013). Experimental research of thermal-oxidative aging on the mechanics of aero-nbr. J. Test. Eval..

[83] Dubovský M., Božek M., Olšovský M. (2015). Degradation of aviation sealing materials in rapeseed biodiesel. J. Appl. Polym. Sci..

[84] Hohe J., Neubrand A., Fliegener S., Appel S. (2021). Performance of Fiber Reinforced Materials under Cryogenic Conditions—A Review. Compos. Part A Appl. Sci. Manuf..

[85] Raj A., Alvi S.M.A.A., Islam K., Motalab M., Xu S. (2023). An Atomistic Study of the Tensile Deformation of Carbon Nanotube–Polymethylmethacrylate Composites. Polymers.

[86] Xiong X., Tao X., Zhou Q.J., Li J.X., Volinsky A.A., Su Y.J. (2016). Hydrostatic Pressure Effects on Hydrogen Permeation in A514 Steel during Galvanostatic Hydrogen Charging. Corros. Sci..

[87] Fujiwara H., Nishimura S. (2012). Evaluation of Hydrogen Dissolved in Rubber Materials under High-Pressure Exposure Using Nuclear Magnetic Resonance. Polym. J..

[88] Martin M., Weber S., Theisen W., Michler T., Naumann J. (2011). Effect of Alloying Elements on Hydrogen Environment Embrittlement of AISI Type 304 Austenitic Stainless Steel. Int. J. Hydrogen Energy.

[89] Sun B., Lu W., Gault B., Ding R., Makineni S.K., Wan D., Wu C.-H., Chen H., Ponge D., Raabe D. (2021). Chemical heterogeneity enhances hydrogen resistance in high-strength steels. Nat. Mater..

[90] Anyalebechi P.N. (1995). Analysis of the Effects of Alloying Elements on Hydrogen Solubility in Liquid Aluminum Alloys. Scr. Metall. Et Mater..

[91] Marchi C.S., Somerday B. (2012). Technical Reference for Hydrogen Compatibility of Materials. https://www.osti.gov/servlets/purl/1055634.

[92] Arafin M.A., Szpunar J.A. (2011). Effect of bainitic microstructure on the susceptibility of pipeline steels to hydrogen induced cracking. Mater. Sci. Eng. A.

[93] Srinivasan R., Neeraj T. (2014). Hydrogen embrittlement of ferritic steels: Deformation and failure mechanisms and challenges in the oil and gas industry. JOM.

[94] Michler T., Wackermann K., Metals F.S. (2021). Review and assessment of the effect of hydrogen gas pressure on the embrittlement of steels in gaseous hydrogen environment. Metals.

[95] Ono H., Nait-Ali A., Diallo O.K., Benoit G., Castagnet S. (2018). Influence of pressure cycling on damage evolution in an unfilled EPDM exposed to high-pressure hydrogen. Int. J. Fract..

[96] Sun Y., Lv H., Zhou W., Zhang C. (2020). Research on hydrogen permeability of polyamide 6 as the liner material for type ΙV hydrogen storage tank. Int. J. Hydrogen Energy.

[97] Bandyopadhyay P., Nguyen T., Li X., Kim N.H., Lee J.H. (2017). Enhanced Hydrogen Gas Barrier Performance of Diaminoalkane Functionalized Stitched Graphene Oxide/Polyurethane Composites. Compos. Part B Eng..

